# Multiple mechanisms underlying rectification in retinal cyclic nucleotide-gated (CNGA1) channels

**DOI:** 10.1002/phy2.148

**Published:** 2013-11-22

**Authors:** Manuel Arcangeletti, Arin Marchesi, Monica Mazzolini, Vincent Torre

**Affiliations:** 1 Neuroscience Area, International School for Advanced Studies (SISSA)Trieste, Italy; 2 CBM S.c.r.l., Area Science ParkBasovizza, 34012, Trieste, Italy

**Keywords:** CNG channels, gating currents, voltage sensor

## Abstract

In cyclic nucleotide-gated (CNGA1) channels, in the presence of symmetrical ionic conditions, current–voltage (*I*-*V*) relationship depends, in a complex way, on the radius of permeating ion. It has been suggested that both the pore and S4 helix contribute to the observed rectification. In the present manuscript, using tail and gating current measurements from homotetrameric CNGA1 channels expressed in Xenopus oocytes, we clarify and quantify the role of the pore and of the S4 helix. We show that in symmetrical Rb^+^ and Cs^+^ single-channel current rectification dominates macroscopic currents while voltage-dependent gating becomes larger in symmetrical ethylammonium and dimethylammonium, where the open probability strongly depends on voltage. Isochronal tail currents analysis in dimethylammonium shows that at least two voltage-dependent transitions underlie the observed rectification. Only the first voltage-dependent transition is sensible to mutation of charge residues in the S4 helix. Moreover, analysis of tail and gating currents indicates that the number of elementary charges per channel moving across the membrane is less than 2, when they are about 12 in K^+^ channels. These results indicate the existence of distinct mechanisms underlying rectification in CNG channels. A restricted motion of the S4 helix together with an inefficient coupling to the channel gate render CNGA1 channels poorly sensitive to voltage in the presence of physiological Na^+^ and K^+^.

## Introduction

Ion channels are ubiquitous proteins playing a fundamental role in cellular functions (Hille 1992). They are grouped in superfamilies evolved from a common ancestor (Jan and Jan 1992; Jegla et al. 2009). The superfamily of voltage-gated ion channels comprises Na^+^, K^+^, and Ca^2+^ channels the gating of which – that is, transitions between the open and closed conformation – is highly dependent on the voltage across the membrane, as well as cyclic nucleotide-gated (CNG) channels where the gating is primarily controlled by binding of cyclic nucleotides (CNs) (Jan and Jan 1992; Yu et al. 2005; Jegla et al. 2009).

Voltage-dependent gating in native channels and both in WT and pore mutant CNG channels have been studied, demonstrating a clear coupling between voltage gating and channel activation by cGMP (Karpen et al. 1988; Benndorf et al. 1999; Nache et al. 2006; Martínez-François et al. 2009, 2010). The current rectification observed at low cGMP concentration becomes negligible at saturating concentration of agonist and is attributed to an increase in the open probability (Karpen et al. 1988; Benndorf et al. 1999). The molecular origin underpinning this moderate voltage sensibility is still poorly understood and is possibly caused by the mobility of the selectivity filter (Nache et al. 2006; Martínez-François et al. 2009).

Up until recent years, it was believed that at saturating concentration of agonist, the gating of CNG channels was poorly controlled by voltage. This view was based on recordings of macroscopic currents and single-channel openings obtained in the presence of Li^+^, Na^+^, and K^+^. We have recently shown that in the presence of larger alkali monovalent cations, such as Rb^+^ and Cs^+^ and of organic cations such as methylammonium (MA^+^) and dymethylammonium (DMA^+^), gating of CNGA1 channels is also powerfully controlled by voltage (Marchesi et al. 2012). If at positive voltages *V* the open probability *P*_o_ is higher than at negative voltages such as at −200 mV, large tail currents *I*_t_ are expected to be measured at −200 mV when they are preceded by steps at positive voltages *V*. In fact, ion channels which are open at positive voltages will close with delays when *V* returns to −200 mV, giving raise to *I*_t_.

In the present manuscript, we aim to dissect the mechanisms underlying the observed rectification and to clarify the role of the S4 helix by analyzing *I*_t_ and gating currents (*I*_*g*_) in the presence of alkali monovalent and a variety of organic cations. We show that at saturating cGMP (i) in symmetrical Rb^+^ and Cs^+^ open-pore rectification prevails in macroscopic currents, whereas voltage-dependent gating becomes larger in symmetrical EA^+^ and DMA^+^ where the open probability at positive voltages can be 10–20 times larger than at negative ones; (ii) the analysis of tail currents in DMA^+^ suggests that at least two voltage-dependent reactions are at the basis of the observed rectification with an apparent valence (*z*) of 1.70 ± 0.10 and 1.00 ± 0.13, respectively; (iii) neutralization of the second arginine in the S4-transmembrane segments significantly affects only the steeper voltage-dependent transition; (iv) *I*_*g*_ measurements from giant patches containing more than 20,000 CNGA1 channels indicate that the number of elementary charges *z* moving per channel is not larger than 2.

## Material and Methods

### Ethical approval

All the studies have been approved by the SISSA's Ethics Committee according to the Italian and European guidelines for animal care (d.l. 116/92; 86/609/C.E.). All *Xenopus laevis* surgeries were performed under general anesthesia and using aseptic technique. Anesthesia was obtained by immersion in a 0.2% solution of Tricaine methane sulfonate (MS-222) for 15–20 min. The individual donor animals were used up to five times. A minimum of 1-month recovery period was ensured between ovarian lobe resection from the same animal to avoid distress.

### Molecular biology

The CNGA1 channel from bovine rod consisting of 690 amino acids was used. Tandem dimer constructs were generated by the insertion of one copy of the CNGA1 DNA into a vector pGEMHE already containing another copy of CNGA1 DNA. At the end of the cloning process, two copies of the CNGA1 DNA were connected by a 10-amino acid linker, GSGGTELGST, joining the C terminus of the first CNGA1 with the N terminus of the second one. This second subunit was made by replacing the ApaI restriction site GGGCCC at the end of the CNGA1 DNA without changing the amino acid GGTCCC and adding to the start codon a new ApaI restriction site, followed by a linker using a PCR reaction. Subunits were linked after HindIII/ApaI was cut. cDNAs were linearized and were transcribed to cRNA in vitro using the mMessage mMachine kit (Ambion, Austin, TX). The RxQ and RxQ_WT nomenclature in the text refers to homotetramers and tandem dimer constructs, respectively. Rx indicates one of the first four Arginines in the S4 helix, where R1, R2, R3, and R4 refer to Arg269, Arg272, Arg275, and Arg278, respectively.

### Oocyte preparation and chemicals

Mutant channel cRNAs were injected into *X. laevis* oocytes (“Xenopus express” Ancienne Ecole de Vernassal, Le Bourg 43270, Vernassal, Haute-Loire, France). Oocytes were prepared as described by Nizzari et al. (1993). Injected eggs were maintained at 18°C in a Barth solution supplemented with 50 μg/mL of gentamicin sulfate and containing (in mmol/L): 88 NaCl, 1 KCl, 0.82 MgSO_4_, 0.33 Ca(NO_3_)_2_, 0.41 CaCl_2_, 2.4 NaHCO_3_, 5 TRIS-HCl (Tris (hydroxymethyl)aminomethane hydrochloride), pH_o_ 7.4 (buffered with NaOH). During the experiments, oocytes were kept in a Ringer solution containing (in mmol/L): 110 NaCl, 2.5 KCl, 1 CaCl_2_, 1.6 MgCl_2_, 10 HEPES-NaOH, pH_o_ 7.4 (buffered with NaOH). Usual salts and reagents were purchased from Sigma Chemicals (St. Louis, MO).

### Recording apparatus

cGMP-gated currents from excised patches (Hamill et al. 1981) were recorded with a patch-clamp amplifier (Axopatch 200, Axon Instruments Inc., Foster City, CA), 2–6 days after RNA injection at room temperature (20–24°C). The perfusion system was as described by Sesti et al. (1995) and allowed a complete solution change in less than 1 sec. Macroscopic and single-channel current recordings obtained with borosilicate glass pipettes had resistances of 2–5 MΩ in symmetrical standard solution. The standard solution on both sides of the membrane consisted of (in mmol/L) 110 NaCl, 10 HEPES (4-(2-Hydroxyethyl)piperazine-1-ethanesulfonic acid), and 0.2 ethylenediaminetetraacetic acid (pH_o_ 7.4). When the cation X^+^ was used as the charge carrier, NaCl in the standard solution on both sides of the membrane patch was replaced by an equimolar amount of the cation X^+^ (buffered at pH_o_ 7.4 with tetramethylammonium hydroxide). We used Clampex 10.0, Clampfit 10.1, SigmaPlot 9.0, and MatLab 7.9.0 for data acquisition and analysis. Gating currents *I*_*g*_ were measured from excised patches containing at least 10^4^ channels both in the absence and presence of 1 mmol/L cGMP with 110 mmol/L TEACl (tetraethylammonium chloride) or NMDG (N-Methyl-D-Glucamine) on both sides of the membrane patch (Perozo et al. 1992; Seoh et al. 1996). In the absence of cGMP, similar results were obtained with 110 mmol/L NaCl on both sides of the patch. Each trace represents the mean of 20 recordings. Patch pipettes have a resistance of 0.2–1 MΩ and a diameter of 10–20 *μ*m (Hilgemann and Lu 1998) as determined from the internal glass opening by visual inspection under a 40× light microscope after fire polishing. Capacitive currents were reduced by parafilm–oil mixture (Hilgemann and Lu 1998) and the P/−4 protocol (Armstrong and Bezanilla 1974). If not otherwise indicated, traces were low pass filtered at 10 kHz and current signals were sampled with a 16-bit A/D converter (Digidata 1440A; Axon Instruments), using a sampling rate of 50 kHz.

### Data analysis

Normalized tail currents *I*_t_−*I*_min_/*I*_+200_ against voltage relationship in the presence of symmetrical DMA^+^ were fitted with a two components Boltzmann function:


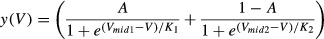
(1)

where *A* is a scaling factor for the first Boltzmann component; *V*_mid*i*_ and *k*_*i*_ are the voltages that give the half-maximal activation and the slope factors for the *i* Boltzmann component, respectively. The gating charge associated with each Boltzmann component *z*_*i*_ can be obtained using *z*_*i*_ = R*T*/*k*_*i*_F, where *T* = 295 K, and R and F are the gas and the Faraday constants, respectively.

If not otherwise indicated data are presented as mean ± SEM, with n indicating the number of patches. Statistical significance for parametric analysis was determined using unpaired two-tailed *T*-test or single-variable analysis of variance (ANOVA), as indicated. For pairwise comparisons, Bonferroni test was used as post hoc test. A value of *P* < 0.01 was considered significant. Data analysis and figures were made with Clampfit 10.1 (Molecular Devices, Sunnyvale, CA), Sigmaplot 12.0 (Systat Software, Chicago, IL) and MatLab 7.9.0 (MathWorks, Natick, MA).

### Counting the number N of CNGA1 channels in excised giant membrane patches

**Figure 1:**
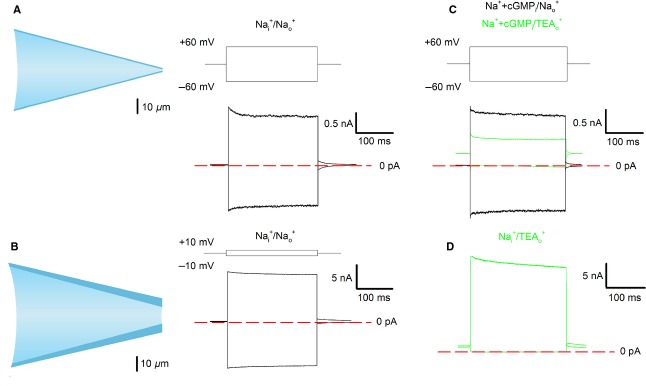
Counting the number of channels in excised giant membrane patches. (A) Currents recorded at ±60 mV in the presence of 1 mmol/L cGMP in symmetrical 110 mmol/L NaCl using patch pipette with a diameter of 1–2 *μ*m. (B) Currents recorded at ±10 mV in the presence of 1 mmol/L cGMP in symmetrical 110 mmol/L NaCl using patch pipette with a diameter of 12 *μ*m. (C) blockage by extracellular TEACl in outside-out patches. Black traces represent currents at ±60 mV in symmetrical 110 mmol/L NaCl and in green the same but with TEACl in the bath solution and NaCl + 1 mmol/L cGMP inside the patch pipette. (D) Currents recorded at ±60 mV in the presence of 1 mmol/L cGMP with 110 mmol/L NaCl in bath solution and 110 mmol/L TEACl inside a large patch pipette. A, B, and D are excised patches in inside-out configuration, whereas C is in an outside-out patch. Red broken lines indicate 0 current level.

A reliable measurement of *I*_*g*_ can be obtained only when excised giant membrane patches contain at least 10^4^ channels as in experiments using cut-open voltage-clamp (Perozo et al. 1992) or in macro/giant membrane patches (Hilgemann and Lu 1998). In the presence of 1 mmol/L cGMP, the open probability *P*_o_ at +60 mV of CNGA1 channels is 0.8 ± 0.08 (*n* = 3) and the single-channel conductance *γ*_sc_ is 30 ± 3 pS (*n* = 3). The number *N* of CNGA1 channels is estimated as:


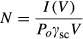
(2)

where *I* is the macroscopic current measured at membrane voltage (*V*). With patch pipettes of the diameter of 1–2 −μm, the current I at ±60 mV could be 1 or 2 nA (Fig. 1A) corresponding to values of *N* from 0.7 ± 0.1 to 1.4 ± 0.3 × 10^3^. Using pipettes of a diameter varying between 10 and 20 μm, the amplitude of I at ±10 mV could be ≥10 nA (Fig. 1B) indicating that *N* ≥ 4.2 ± 0.8 × 10^4^. When 110 mmol/L NaCl in the patch pipette was substituted entirely with TEACl – impermeable ions – for measuring *I*_*g*_, equation 2 was substituted with:


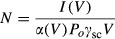
(3)


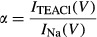
(4)

where *α* is the fractional blockage of the Na^+^ current caused by TEACl. As shown in Figure 1C, when extracellular Na^+^ was substituted with TEA^+^ in outside-out patches at +60 mV *α* = 0.48 ± 0.04 (*n* = 4). In the presence of 110 mmol/L TEACl in the patch pipette and 110 mmol/L NaCl in the bathing medium, a cGMP-gated current of 20 nA was recorded at + 60 mV (Fig. 1D). In these experiments, the estimated value of *N* from equation (4) was 2.9 ± 0.8 × 10^4^.

### Properties of gating currents of spHCN channels measured with giant membrane patches

**Figure 2:**
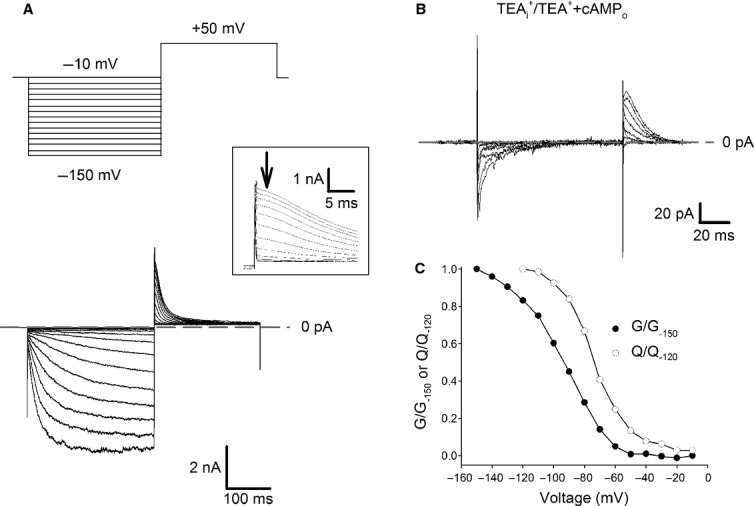
Comparison of the dependence of *G*/*G*_−150_ and *Q*/*Q*_−120_ on voltage in the spHCN channels. (A) Current recordings from giant patches excised from oocytes injected with the mRNA coding for the spHCN channels (lower panel). Voltage steps are shown in the upper panel. Current recordings obtained in the presence of 1 mmol/L cAMP and in symmetrical 110 mmol/L KCl conditions. The inset in the box represents the tail current (tail potential at +50 mV) and the arrow corresponds to the isochronal currents used to calculate the conductance (G). (B) Gating current for spHCN channel in response to voltage steps from −10 to −120 mV, from a holding potential of −10 mV, tail potential +50 mV. (C) Ionic conductance (*G*/*G*_−150_ filled circles) and normalized charge movement (*Q*/*Q*_−120_ open circles) for the electrical recordings in A and B; the conductance (G) was calculated from tail current (arrow in panel A) normalized as *G*(*V*) = [*I*(*V*)−*I*_min_]/(*I*_max_−*I*_min_); the gating current (Fig. 2A) was integrated in time so to obtain the charge (*Q*). Gray broken lines in panels A and B indicate 0 current level.

In order to verify that our experimental conditions could measure gating currents in a reliable way, we expressed in oocytes the mRNA coding for Hyperpolarization-activated cyclic nucleotide-gated (HCN) channels from the sea urchin sperm (spHCN). Gating currents from these channels were already measured with cut-open oocyte technique (Männikkö et al. 2002). The comparison with already published data and those obtained by us with giant membrane patches represents a good test of our experimental setup. The activation curve in the spHCN channel was determined from the isochronal tail current analysis at 50 mV following voltage steps from −10 mV to −150 mV in the presence of 1 mmol/L cAMP (Fig. 2A). Gating currents were obtained in response to voltage steps from −10 to −120 mV, from a holding potential of −10 mV, tail potential +50 mV (Fig. 2B). Figure 2C reproduces the relationship between *G*/*G*_−150_ (filled symbols) and *V* in symmetrical 110 mmol/L KCl conditions and the relation between *Q*/*Q*_−120_ (open symbols) and *V* in symmetrical 110 mmol/L TEACl conditions. The total charge *Q* at a given voltage *V*, *Q*(*V*), was obtained by integrating the off-gating current (i.e., the current associated with the movement of the voltage sensor from the activated to the resting state) over time.

### Membrane fluctuations

In the presence of 1 mmol/L cGMP, WT CNGA1 channels have a dominant single-channel conductance. Under these conditions, the noise variance (*σ*^2^) is related to the amplitude of the mean current (*I*) by the following equation:


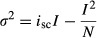
(5)

where *i*_sc_ is the amplitude of the single-channel current and *N* is the number of channels present in the membrane patch (Neher and Stevens 1977). At hyperpolarized membrane potentials, when voltage drives protons into the channel pore, a fast mechanism of blockage could result in an increased open-channel noise (*σ*_o_^2^) from fast, nonresolved gating transitions (Root and MacKinnon 1994). Therefore, we ask whether the open-channel noise could contribute to the differences in noise level here described. If *σ*_e_^2^ is the ensemble variance of the open-pore fluctuations and *σ*^2^ is the noise associated with channel opening and closing, the overall amplitude of membrane fluctuations is expected to be:


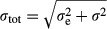
(6)

where 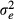 can be calculate as:


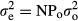
(7)

The overall contribution of *σ*_e_ to *σ*_tot_ depends only on two independent parameters: *σ*_o_ /*i*_sc_ and *P*_o._ At +100 mV *σ*_o_/*i*_sc_ is around 0.1, and does not depend on the permeant ion (Kusch et al. 2004). If Cs^+^ permeation is considered, at −200 mV *P*_o_ has to be less than 0.5 (Marchesi et al. 2012). Even in the unlikely scenario where *σ*_o_/*i*_sc_ degrades to 0.5, *σ*_e_ is not expected to increase membrane fluctuations more than 25%, while the differences in noise levels experimentally observed between +200 and −200 mV are usually between 700 and 1000%. It is fair to conclude that the open-channel noise is not likely to explain most of the differences observed in the noise level in the present manuscript.

### Molecular modeling

Sequences alignment were performed using The UniProt Consortium server-based software (available at http://www.uniprot.org/) and further visualized and edited with Jalview 2.8 (Clamp et al. 2004). Pore cartoons were prepared using known structures of K_v_ and NaK chimeric channels available in the Protein Data Bank (PDB) using the DeepView module of the Swiss-PDBViewer (v4.04) software (Guex and Peitsch 1997).

## Results

### Ionic permeation of alkali monovalent cations through WT CNGA1 channels

**Figure 3:**
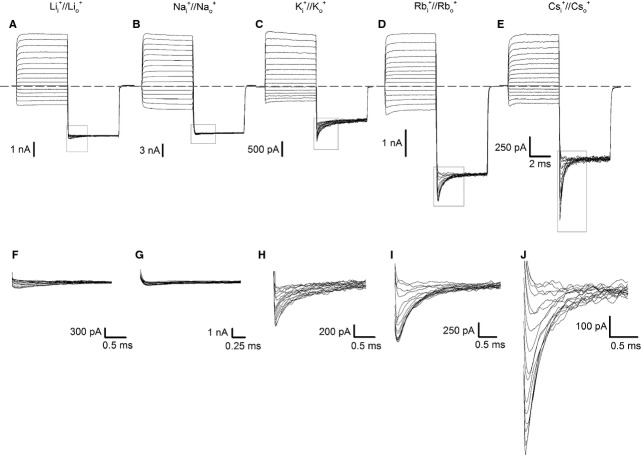
Macroscopic and tail currents in symmetrical Li^+^, Na^+^, K^+^, Rb^+^, and Cs^+^ conditions. (A–E) Macroscopic currents recorded from excised patches in symmetrical solutions of Li^+^ (A), Na^+^ (B), K^+^ (C), Rb^+^ (D), and Cs^+^ (E) with 1 mmol/L cGMP in the intracellular medium. Leak and capacitative components were removed subtracting from the cGMP-activated current those records obtained in response to the same voltage protocol but without cGMP. The voltage commands were stepped from a holding potential of 0 mV to prepulses varying between −100 and +200 mV in 20 mV steps. At the end, the voltage command was moved to −200 mV for 5 msec in order to elicit tail currents *I*_t_(*V*). Gray broken line indicates 0 current level. (F–J), Enlargement of tail currents (boxed areas in A–E) in Li^+^ (F), Na^+^ (G), K^+^ (H), Rb^+^ (I), and Cs^+^ (J). Current recordings were filtered at 10 kHz and sampled at 50 kHz to resolve rapid transients.

Current recordings obtained under voltage clamp in symmetrical conditions of Li^+^, Na^+^, K^+^, Rb^+^, and Cs^+^ (110 mmol/L) are shown in Figure 3.

**Figure 4:**
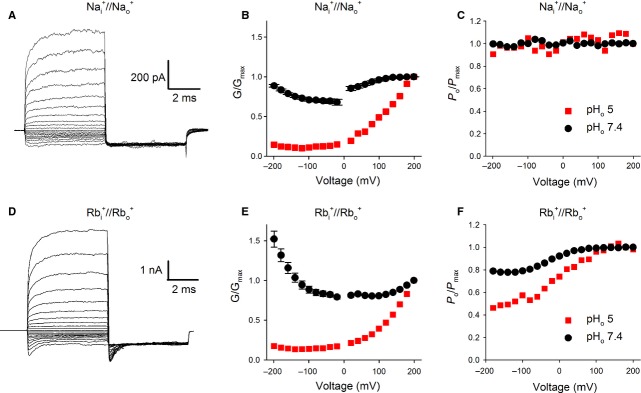
Macroscopic and tail currents in symmetrical Na^+^ and Rb^+^ conditions at low pH_o_. (A) Macroscopic currents recorded from excised patches in symmetrical solutions of Na^+^ with 1 mmol/L cGMP on the cytoplasmic side and pH_o_ 5 at the extracellular side of the membrane (pH_i_ = 7.4). Tail currents at −200 mV evoked by prepulses at voltages from −200 to +200 mV (ΔV=20 mV); current recordings were filtered at 10 kHz and sampled at 50 kHz to resolve rapid transients. (B) Dependence of *G*/*G*_+200_ on V for Na^+^ at pH_o_ 7.4 (black dots) and 5 (red squares). (C) Estimation of *P*_o_/*P*_o_max_ from tail currents. *P*_o_/*P*_o_max_ was estimated as *I*_t_/*I*_t_max_. (D) as in A but in symmetrical solutions of Rb^+^. E, as in B but for Rb^+^. F, as in C but for Rb^+^.

The shape of these recordings differs – according to the permeating ion – in several aspects. First, the current–voltage *I*-*V* relationship depends on the permeant ion, being outwardly rectifying in the presence of K^+^ (Fig. 3C), almost linear in the presence of Li^+^ and Na^+^ (Fig.3A and B), and inwardly rectifying in the presence of Rb^+^ and Cs^+^ (Fig. 3D and E). Second, small tail currents I_t_ can be observed in the presence of small alkali cations, such as Li^+^, Na^+^, and K^+^ (Figs. 3A–C and F–H) which become significant with the larger alkali cations Rb^+^ and Cs^+^ (Fig. 3D, E, I, and J). These transient currents are not likely to arise from a voltage-dependent proton blockage (Root and MacKinnon 1994), as very similar tail currents were observed when extracellular pH_o_ was lowered to 5 (Fig. 4). In fact, Na^+^ and Rb^+^ currents observed at −200 mV increase from 5 to 10 times when proton concentration is decreased from pH_o_ 5 to 7.4 (Fig. 4A, B and D, E for Na^+^ and Rb^+^ conditions, respectively), reflecting proton blockage. However, only a modest effect on *P*_o_/*P*_o_max_ curves was observed (Fig. 4C and F), suggesting that tail currents do not arise from Glu363 protonation/deprotonation. These recordings suggest that a fast mechanism of blockage underscore proton action in CNGA1 channels, which is not expected to result in significant measurable tail currents within the used recording bandwidth (Fig. 4). Thus, the observed current decays in response to voltage jumps are likely to reflect a voltage-dependent conformational change.

**Figure 5:**
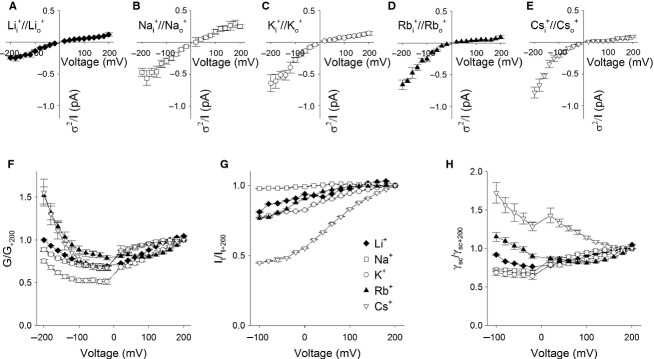
*G*/*G*_+200_, *P*_o_/*P*_o+200_, *γ*/*γ*_+200_ against V relationships and noise analysis in symmetrical Li^+^, Na^+^, K^+^, Rb^+^, and Cs^+^ conditions. (A–E) Relationship between *σ*_I_^2^/*I* and V for Li^+^, (A), Na^+^ (B), K^+^ (C), Rb^+^ (D), and Cs^+^ (E). The background noise observed in the absence of cGMP was subtracted; (*n* ≥ 6). F, Dependence of *G*/*G*_+200_ on *V* for Li^+^, Na^+^, K^+^, Rb^+^, and Cs^+^. G/G_+200_ is the fractional steady conductance obtained from macroscopic currents elicited by voltage commands from −200 to +200 mV (Δ*V* = 20 mV). G, Dependence of *P*_o_/*P*_o+200_ on V for Li^+^, Na^+^, K^+^, Rb^+^, and Cs^+^ obtained from tail currents as *I*_t_/*I*_t+200_. (H) Dependence of *γ*_sc_/*γ*_sc+200_ on *V* for Li^+^, Na^+^, K^+^, Rb^+^, and Cs^+^ ions. The *γ*_sc_/*γ*_sc+200_ relationship for various ions was computed from the *G*/*G*_+200_ and *P*_o_/*P*_o+200_ plots as described in the text. Li^+^ (filled diamonds), Na^+^ (open squares), K^+^ (open circles), Rb^+^ (filled triangles), and Cs^+^ (open triangles); (*n* ≥ 4).

If these current recordings are not averaged over several trials, another remarkable and unexpected feature is observed: in the presence of Rb^+^ and Cs^+^ current recordings at negative voltages are significantly noisier than those at positive voltages, although the mean outward and inward currents are approximately similar (Marchesi et al. 2012). The relation *σ*^2^/*I* against *V* for different ions, where *σ*^2^ is the current variance and I the macroscopic mean current, is shown in Figure 5A–E. If channels open to a single conductance level, *σ*^2^/*I* is equal to *i*_sc_(1-*P*_o_), where i_sc_ is the single-channel current and *P*_o_ is the open probability (Neher and Stevens 1977). As the open-channel noise associated with the proton blockage at resting membrane potentials is not expected to contribute significantly to the differences observed in membrane fluctuations (see Methods), if both, *i*_sc_ and *P*_o_ do not depend significantly on voltage the *σ*^2^/*I* versus voltage relationship is expected to be approximately linear. While this seems to be so for Li^+^ and Na^+^ permeation (5A, B), it is not so when K^+^, Rb^+^, and Cs^+^ (5C–E) are used as the charge carriers, suggesting that in the presence of symmetrical Rb^+^ and Cs^+^ – and, to a lesser extent, also in the presence of K^+^ – WT CNGA1 channels have a voltage-dependent gating (Marchesi et al. 2012).

How do voltage-dependent changes in *i*_sc_ and *P*_o_, as suggested from noise analysis and single-channel recordings (Marchesi et al. 2012), relate to the *I*–*V* relationship shown in Figure 3, and how do they depend on the permeant ion? To answer this basic question, we have obtained and compared the dependence from *V* of the normalized conductance *G*/*G*_+200_ (Fig. 5F) and the amplitude of the normalized tail current *I*_t_/*I*_t+200_ (Fig. 5G). The *I*_t_(*V*)/*I*_t+200_ relationship provides a good estimate of the dependence of *P*_o_/*P*_o+200_ on *V*, whereas *G*(*V*)/*G*_+200_ depends on both, *γ*_sc_ and *P*_o_. From the knowledge of *I*_t_(*V*)/*I*_t+200_ and *G*(*V*)/*G*_+200_ the dependency of the relative single-channel conductance *γ*_sc_/*γ*_sc+200_ on *V* was inferred by dividing the values of *G*/*G*_+200_ obtained at different voltages by the corresponding values of *P*_o_/*P*_o+200_. The *γ*_sc_(*V*)/*γ*_sc+200_ relationship thus obtained is shown in Figure 5H and illustrates the dependency of the open-pore rectification on the permeant ions.

**Figure 6:**
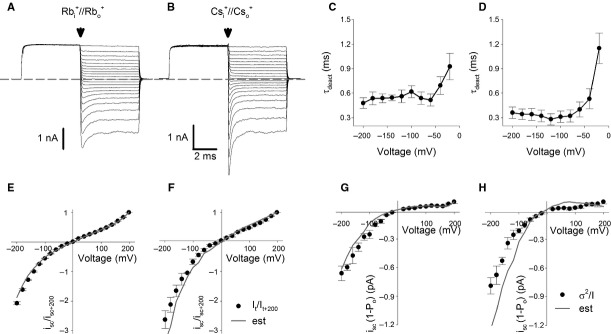
Tail currents, *i*_sc_ /*i*_sc+200_ against *V* relationship and predicted macroscopic current noise in symmetrical Rb^+^ and Cs^+^ conditions. (A,B) Macroscopic currents in symmetrical solutions of Rb^+^ (A) and Cs^+^ (B). Voltage prepulse held at +200 mV was followed by test potentials ranging from −200 mV to +200 mV (Δ*V* = 20 mV). Gray broken line indicates 0 current level. (C,D) Dependence of inward transient kinetics on *V* in symmetrical Rb^+^ (C) and Cs^+^ (D). These transients decay with a single time constant between 0.3 and 1.2 msec. (E,F) Dependence of *i*_sc_ /*i*_sc+200_ on *V* for Rb^+^ (E) and Cs^+^ (F) obtained from instantaneous *I*–*V* relationship measured immediately after the depolarizing voltage prepulse (see arrows in A,B). Solid gray lines represent the expected *i*_sc_ /*i*_sc+200_ curves from the *γ*_sc_ /*γ*_sc+200_ plots in 5H; (*n* ≥ 4). G,H, Dependence of i_sc_(1-*P*_o_) on *V* for Rb^+^ (G) and Cs^+^ (H). In the presence of 1 mmol/L cGMP, WT CNGA1 channels have a dominant single-channel conductance (*γ*_sc_). Under these conditions, the noise variance (*σ*_I_^2^) is related to the amplitude of the mean current (I) by: *σ*_I_^2^ = *i*_sc_*I*−*I*^2^/*n*. If *i*_sc_ is the single-channel current then the ratio *σ*_I_^2^/*I* of the variance of current fluctuations (*σ*_I_^2^) and of the mean current (*I*) is *i*_sc_(1−*P*_o_). Filled circles show the experimentally observed noise (*σ*_I_^2^/*I*) in macroscopic currents at different voltages (see also Fig. 5A–E). Solid lines represent the expected *i*_sc_(1−*P*_o_) curves computed from the *P*_o_/*P*_o+200_ and *i*_sc_ /*i*_sc+200_ plots as described in the text.

Alternatively, when clear tail currents are observed as in the presence of Rb^+^ and Cs^+^, the dependency of *i*_sc_/*i*_sc+200_ and of deactivation time constant (*τ*_deact_) from V could be directly measured from a different voltage protocol (Fig. 6A and B). The voltage commands were first stepped from a holding potential of 0 mV to a prepulse at 200 mV to maximally open channels and were next followed by test voltages varying between −200 and 200 mV in 20 mV steps to induce tail currents. As expected – reflecting a voltage-dependent transition – the deactivation time constants are voltage dependent, being faster at hyperpolarized potentials, either when Rb^+^ (Fig. 6C) or Cs^+^ (Fig. 6D) ions are used as the charge carriers. The normalized *I*–*V* relationship obtained by plotting the instantaneous tail currents measured at the beginning of each step (see arrows in Fig. 6A and B) versus the step voltage is shown in Figure 6E and F for Rb^+^ and Cs^+^, respectively (filled circles). These relationships are inwardly rectifying, and are almost identical to the relationship estimated for the *γ*_sc_(*V*)/*γ*_sc+200_ shown in Figure 5H (compare solid lines and filled circles in Fig. 6E and F). These results demonstrate that in symmetrical Rb^+^ and Cs^+^ the open-channel current strongly depends on *V*.

To double check the accuracy of our *P*_o_(*V*)/*P*_o+200_ and *i*_sc_(*V*)/*i*_sc+200_ plots, we asked whether it was possible to recapitulate the *σ*^2^/*I*(*V*) relationship obtained from noise analysis. First, the *i*_sc_(*V*)/*i*_sc+200_ plots determined from tail currents (Fig. 6E and F) were scaled to the *i*_sc_ experimentally measured from single-channel recordings at +160 mV in symmetrical Rb^+^ and Cs^+^ (Marchesi et al. 2012) in order to obtain the *i*_sc_ versus *V* relation. The *P*_o_/*P*_o+200_ relationships were also scaled to the absolute *P*_o_ at +160 mV determined from noise analysis as 1 – [(*σ*^2^/*I*)/*i*_sc_] in order to avoid the typical variability inherent to single-channel measurements. *σ*_I_^2^/*I* where then computed from the single-channel parameters as *i*_sc_(1-*P*_o_). Figure 6G and H illustrate the *σ*^2^/*I*(*V*) relationship thus extrapolated in symmetrical Rb^+^ and Cs^+^ which closely tracks the *σ*^2^/*I* relationship experimentally measured in Rb^+^ and – to a lesser extent – in Cs^+^. The discrepancies observed in symmetrical Cs^+^ between the observed and the predicted noise are probably due to the flickering openings and the subconductance states previously reported at hyperpolarized potentials during Cs^+^ permeation (Marchesi et al. 2012).

These observations are consistent with significant differences in both, the single-channel conductance *γ*_sc_ and the open probability *P*_o_ at positive and negative voltages. In the presence of Rb^+^ and Cs^+^ γ_sc_ increases of ∼200% and ∼300% from 200 to −200 mV (Fig. 6E and F), whereas *P*_o_ decreases of ∼25% and ∼55% (Fig. 5G), respectively, resulting in an inwardly rectifying *I*–*V* relationship. When Li^+^, Na^+^, and K^+^ are the charge carriers, the dependency of *γ*_sc_ and *P*_o_ on *V* is very mild (Fig. 5G and H), and the resulting *I*–*V* relationship is almost linear.

Take together, these data clearly demonstrate that the *G*(*V*)/*G*_+200_, *I*_t_(*V*)/*I*_t+200_, and *γ*_sc_(*V*)/*γ*_sc+200_ relationship depend on the ionic species, showing a profound coupling between permeation and gating in CNGA1 channels.

### Ionic permeation of organic cations through WT CNGA1 channels

**Figure 7:**
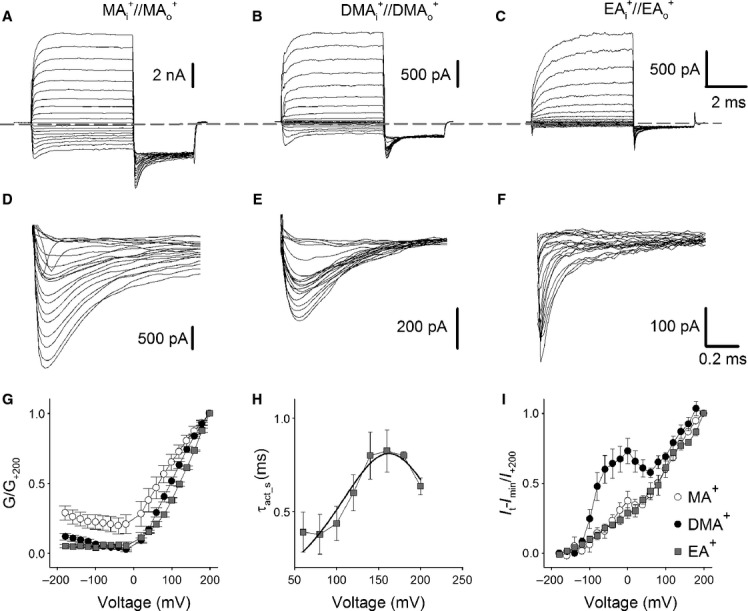
Macroscopic and tail currents in symmetrical MA^+^, DMA^+^, and EA^+^ conditions. (A–C) Macroscopic currents recorded from excised patches in symmetrical solutions of MA^+^ (A), DMA^+^ (B), and EA^+^ (C) with 1 mmol/L cGMP in the intracellular medium. Voltage prepulses from −180 to +200 mV (Δ*V* = 20 mV) were followed by a tail potential at −200 mV. Gray broken line indicates 0 current level. (D–F) Enlargement of tail currents in MA^+^ (D), DMA^+^ (E), and EA^+^ (F). Current recordings were filtered at 10 kHz and sampled at 50 kHz. (G) Dependence of *G*/*G*_+200_ on V for MA^+^, DMA^+^, and EA^+^. MA^+^ open circles, DMA^+^ filled circles, EA^+^ filled squares. (H) Dependence of the slow activation time constants (*τ*_act_s_) on V in the presence of EA^+^. The bell-shaped distribution of *τ*_act_s_ was fitted to the following relation: *τ*_act_s_ = 1/[*α*(*V*)+*β*(*V*)], where *α*(*V*) and *β*(*V*) are the voltage-dependent forward and backward rate constants, respectively. Dependence of *I*_t_−*I*_min_/*I*_+200_, obtained from tail currents, on *V* for MA^+^, DMA^+^, and EA^+^. Symbols as in G; (*n* ≥ 4).

To further explore the linkage between voltage gating and permeation, we analyzed tail currents also in the presence of large organic cations such as MA^+^, DMA^+^, and EA^+^. Figure 7 illustrates macroscopic current recordings obtained in the presence of symmetrical MA^+^ (Fig. 7A), DMA^+^ (Fig. 7B), and EA^+^ (Fig. 7C) from voltage commands very similar to those described in Figure 3. The *I*–*V* relationships were outwardly rectifying for all three cations, being |*I*_+200_/*I*_−200_| equal to 3.31 ± 0.33, 7.22 ± 1.05, and 23.25 ± 0.89 for MA^+^, DMA^+^, and EA^+^, respectively (Fig. 7G), in agreement with previous findings for MA^+^ and DMA^+^ (Marchesi et al. 2012). Moreover, the macroscopic current traces shown in Figure 7A–C show a time-dependent increase in current amplitude at depolarizing potentials. We have analyzed the activation time constant (*τ*_act_) in the presence of EA^+^, where it appears to be particularly slow (Fig. 7C). The time courses could be described as the sum of two exponentials, yielding a fast (*τ*_act_f_) and slow (*τ*_act_s_) time constant. *τ*_act_f_ develops in <100 *μ*s and could not be reliably solved and measured within the used recording bandwidth; however, the dependency of *τ*_act_s_ on V could be studied. The *τ*_act_s_(*V*) relationship appears to be bell shaped (Fig. 7H), indicating that the slower activation component *τ*_act_s_ is associated with the translocation of 0.88 equivalent charges.

Furthermore the analysis of tail currents reveals an unconventional behavior (Fig. 7D–F). In the presence of DMA^+^ (Fig. 7E), and – to a lesser extent – also in the presence of MA^+^ (Fig. 7D) and EA^+^ (Fig. 7F), tail currents exhibit a plateau before further increasing with the prepulse amplitude. These observations are reflected in the prominent hunch observed around 0 mV for DMA^+^ in the *I*_t_−*I*_min_/*I*_+200_ versus V plot (Fig. 7I, filled circles), indicating the possibility that two different charge systems underlie voltage gating.

The *G*(*V*)/*G*_+200_ relationship for DMA^+^ does not mirror the *I*_t_−*I*_min_/*I*_+200_ versus V plot between −200 and 0 mV (Fig. 7G, filled circles) and unexpectedly, the slope of the *G*(*V*)/*G*_+200_ curve is negative within this voltage range (compare Fig. 7G and 7I, filled circles). Whereas the amplitude of tail currents observed at −200 mV depends only on the *P*_o_ at the preceding voltage commands, and hence the *I*_t_−*I*_min_/*I*_+200_ versus *V* plot depends solely on *P*_o_, the *G*(*V*)/*G*_+200_ relationship depends on both *γ*_sc_ and *P*_o_. It is therefore conceivable that in the *G*(*V*)/*G*_+200_ relationship a nonlinearity of *i*_sc_ effectively covers the early voltage-dependent transitions observed at negative voltages in the *I*_t_−*I*_min_/*I*_+200_ versus *V* plot.

**Figure 8:**
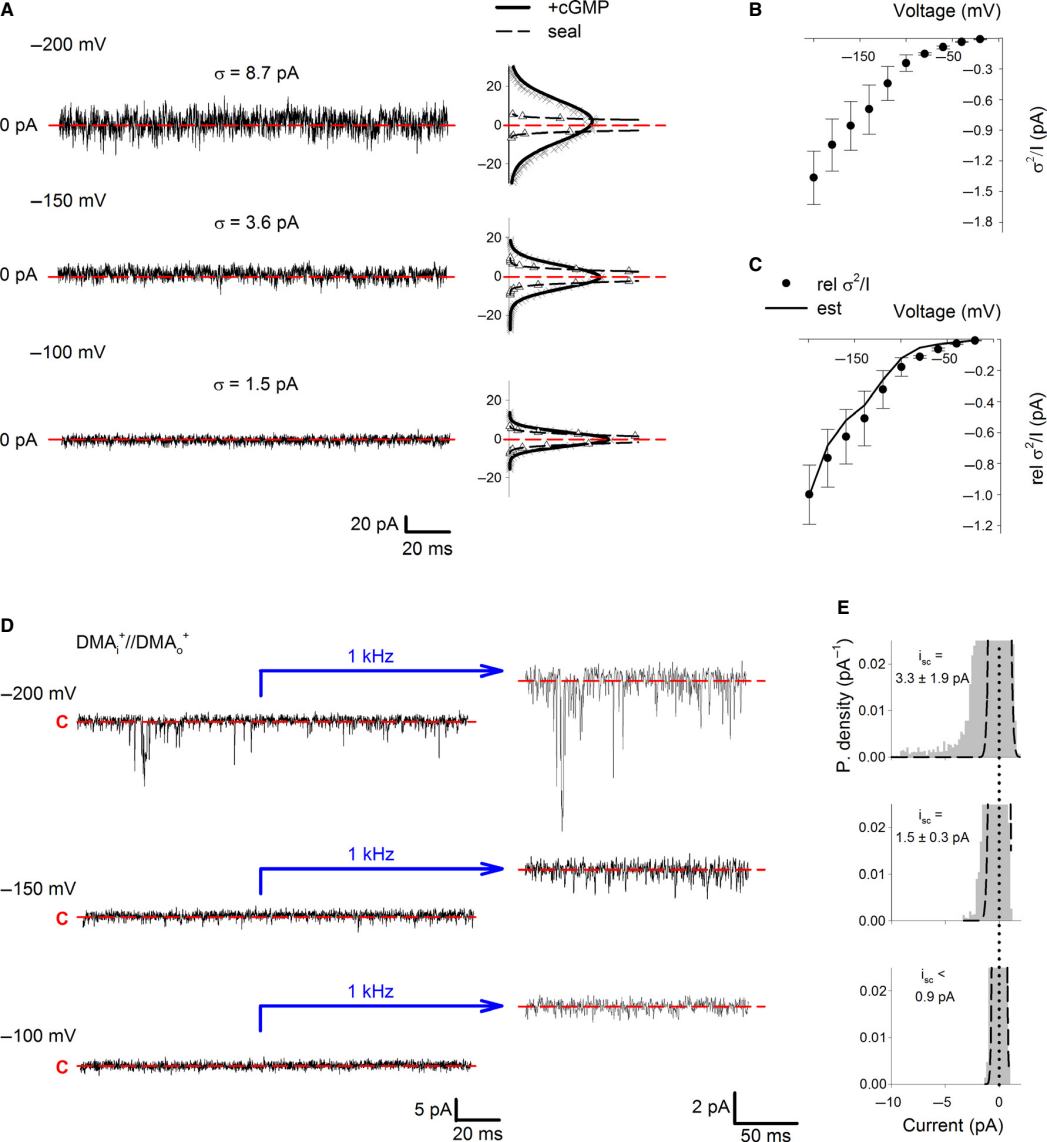
Noise analysis and single-channel recordings in symmetrical DMA^+^ conditions. (A) Macroscopic currents fluctuations in symmetrical solutions of DMA^+^ at −200, −150, and −100 mV from the same excised patch. Current r.m.s. (*σ*) were obtained after background noise subtraction as *σ*_cGMP_ − *σ*_seal_, where *σ*_cGMP_ and *σ*_seal_ indicate the r.m.s recorded in the presence and absence of 1 mmol/L cGMP, respectively. The corresponding current amplitude histogram after mean current subtraction is shown on the right. Crosses and open triangles refer to traces obtained in the presence and absence of cGMP, respectively. Data were fitted with a Gaussian distribution yielding *σ*_cGMP_ and *σ*_seal_ (solid and dashed lines, respectively). Red broken line indicates 0 current level. (B) Relationship between *σ*_I_^2^/*I* and *V* for DMA^+^ (*n* = 3). (C) Relationship between the expected relative single-channel current (*i*_sc_/*i*_sc-200_) and V. Filled circles show the experimentally observed normalized noise (rel *σ*_I_^2^/*I*) at different voltages. Solid line represents the *i*_sc_(*V*)/*i*_sc-200_ curve (est) computed as described in the text. (D) Single-channel recordings at −200, −150, and −100 mV from the same patch in the presence of symmetrical DMA^+^. Current records were acquired at 50 kHz, and filtered at 10 kHz. For presentation purposes, traces were digitally filtered at 5 kHz and 1 kHz (left and right panels, respectively). Dashed lines in single-channel recordings indicate the closed state (C) of the channel. (E) All-point amplitude histograms from recordings as in (D) after offline digital filtering at 1 kHz. Dashed lines refer to all-point amplitude histograms Gaussian fit from records obtained in the same experimental conditions as in (D) but in the absence of cGMP. Single-channel current amplitude that were estimated from the peak current analysis procedure are indicated (Marchesi et al. 2012). The vertical dotted line indicates 0 current level.

In this view, the negative slope of the *G*(*V*)/*G*_+200_ relationship observed between −200 and 0 mV (Fig. 7G) is due to a decrease in single-channel conductance that has overcome the voltage-dependent increase in *P*_o_. This hypothesis leads to the prediction that macroscopic current noise decreases more than linearly with voltage. Figure 8A illustrates current recordings obtained from the same inside-out membrane patch at −200, −150, and −100 mV in the presence of symmetrical DMA^+^. Indeed, current r.m.s. increases more than six times from −100 mV to −200 mV, being 8.7 and 1.5 pA at −200 and −100 mV, respectively (Fig. 8A). Figure 8B shows the *σ*^2^/*I*(*V*) relationship which is equivalent to *i*_sc_(1-*P*_o_) as discussed above. Since between −200 and −100 mV *P*_o_ is almost constant (Fig. 7I), the ratio *σ*^2^/*I* is expected to be driven by changes in the *i*_sc_ within this voltage range. An estimate of the dependency of the relative single-channel current *i*_sc_/*i*_−200_ on *V* was inferred by dividing the values of *G*/*G*_+200_ obtained at different voltages by the corresponding values of *P*_o_/*P*_o+200_. The *i*_sc_(*V*)/*i*_−200_ relationship thus obtained is shown in Figure 8C (solid line) and closely matches the measured normalized noise (filled circles).

**Figure 9:**
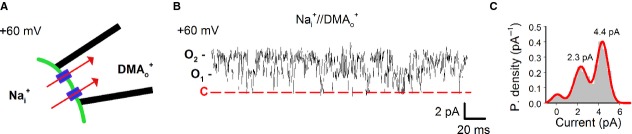
Estimating the number of channels underlying current fluctuations in symmetric DMA^+^ (A) Schematic depicting an excised patch in bi-ionic conditions of intracellular Na^+^ (Na_i_^+^) and extracellular DMA^+^ (DMA_0_^+^) with two CNGA1 channels present in the membrane patch. The arrows indicate the direction of the electrochemical driving force at +60 mV. In bi-ionic conditions, the cGMP-activated current at +60 mV was carried almost by Na^+^ ions moving from the bath solution toward the patch pipette. (B) Single-channel recording from the experimental conditions shown in A. Well-resolved openings slightly larger than those usually observed in symmetrical Na^+^ could be detected. Dashed line indicates the closed (C) state of the channel; O_1_ and O_2_ refer to the two open states. (C) All-point amplitude histogram from the electrical recording shown in B. Data were fitted with a three component Gaussian function (solid red line) indicating that two CNGA1 channels were present in the membrane patch.

To further substantiate the notion that the single-channel conductance is inversely related to the membrane potential at hyperpolarized voltages, we analyzed the properties of single-channel currents in the presence of DMA^+^. Figure 8D and E show electrical recordings obtained from an inside-out patch containing only two CNGA1 channels (Fig. 9) in symmetrical DMA^+^ at −200, −150, and −100 mV. At 5 kHz bandwidth (Fig. 8D, left traces) clear openings of variable amplitude could be observed only at −200 mV. Further filtering at 1 kHz (Fig. 8D, right traces) reveals flickering openings also at −150 mV, but no electrical signal detaching from membrane noise could be reliably measured at −100 mV. In the corresponding amplitude histogram, flickering openings appear as a one-sided tail spreading out from the closed state noise which rapidly reduces as membrane voltage depolarizes (Fig. 8E). Estimates of single-channel currents obtained from peak analysis (Marchesi et al. 2012) show that the single-channel conductance is almost halved from −200 to −100 mV (Fig. 8E).

These results clearly demonstrate that: (i) permeation of large organic cations is associated with outward rectification; (ii) two voltage-dependent transitions are at the basis of the observed voltage gating; (iii) early voltage-dependent transitions occurring at hyperpolarized potentials are covered by the voltage dependency of *γ*_sc_ in the *G*(*V*)/*G*_+200_ relationship.

### The role of positive charges in S4 in voltage sensing

**Figure 10:**
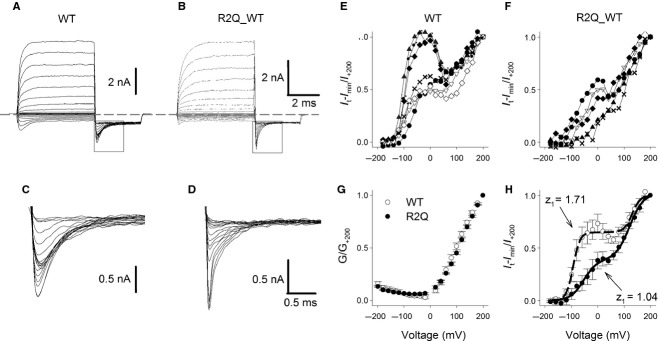
Macroscopic and tail currents in symmetrical DMA^+^ conditions for WT and the R2Q_WT construct. (A, B) Macroscopic currents recorded from excised patches in symmetrical solutions of the DMA^+^ for WT channel (A) and for the R2Q_WT mutant channel (B) with 1 mmol/L cGMP in the intracellular medium. Voltage prepulses from −180 to +200 mV (ΔV = 20 mV) were followed by a tail potential at −200 mV. Gray broken line indicates 0 current level. (C–D) Enlargement of tail currents (boxed areas in A,B) in DMA^+^ for WT channel (C) and for the R2Q_WT mutant channel (D). Current recordings were filtered at 10 kHz and sampled at 50 kH. (E,F) Dependence of *I*_t_−*I*_min_/*I*_+200_ on *V* for DMA^+^ in WT channel (E) and in the R2Q_WT mutant channel (F). Recordings from different patches are shown in different symbols. (G) Dependence of *G*/*G*_+200_ on V in symmetrical DMA^+^ solutions for the WT channel and for the R2Q_WT mutant channel. WT (open circles), R2Q_WT (filled circles); (*n* ≥ 5). (H) Dependence of *I*_t_−*I*_min_/*I*_+200_ on *V* in DMA^+^ for WT channel and for the R2Q_WT mutant channel. WT (open circles), R2Q (filled circles); (*n* ≥ 5). Data were fitted with a modified two components Boltzmann function (see Methods) yielding the following activation parameters: *z*_1_ = 1.71, 1.04; *V*_mid1_ = −93.64, −53.01; *z*_2_ = 1.07, 0.98; *V*_mid2_ = +130.73, +120.37; for the WT and the R2Q_WT channels, respectively.

If the movement of S4 is responsible for voltage sensing, replacement of Arg269, Arg272, Arg275, and Arg278 in S4 (here referred to as R1, R2, R3, and R4) with a neutral amino acid such as Glutamine is expected to alter gating. None of the RxQ mutant channels give rise to appreciable currents when expressed in oocytes; however, clear cGMP currents could be measured from tandem constructs RxQ_WT where one mutant channel was attached to a WT CNGA1 subunit with an appropriate linker (Marchesi et al. 2012). A significant effect was previously reported in the presence of large cations such as Cs^+^ only when Arg272 (R2) of the CNGA1 channels was neutralized. This observation prompted us to analyze voltage gating in the R2Q_WT construct in the presence of symmetrical DMA^+^ where tail current analysis indicates the existence of two voltage-dependent transitions (Fig. 7). Figure 10A and B illustrate current recordings obtained in the presence of symmetrical DMA^+^ for the WT and the R2Q_WT construct, respectively. Current rectification and the *G*(*V*)/*G*_+200_ relationship were very similar for both channels, although not identical (Fig. 10A, B, and G). However, a closer inspection of current traces revealed an altered gating in the R2Q_WT mutant channel: the steady-state current was reached more slowly in the R2Q_WT mutants being the activation time constant at 200 mV equal to 0.3 and 0.5 msec for the WT and R2Q_WT channels, respectively. Figure 10C and D show an enlargement of tail currents observed at −200 mV after prepulses varying between −180 and 200 mV for both channels. In the R2Q_WT construct the early voltage-dependent transition is markedly reduced compared to the WT channel and similar results were obtained from five additional patches (Fig. 10E and F). Fitting the normalized tail currents versus voltage relationship with a two components Boltzmann function (Fig. 10H) provides a lower estimate to the gating charge associated with early (*z*_1_) and late (*z*_2_) voltage-dependent reactions. These results show that neutralization of R2 affects only the steeper early voltage-dependent transition (*z*_1_ equal to 1.70 ± 0.10 and 1.00 ± 0.20 for WT and R2Q_WT constructs, respectively; *T*-test, *P* < 0.01, *n* ≥ 5) and further substantiate the notion that S4 helix contributes to voltage gating in CNG channels. The voltage-dependent activation above 100 mV is very similar in both channels (*z*_2_ equal to 1.00 ± 0.13 and 1.04 ± 0.03 for the WT and R2Q_WT constructs, respectively; *T*-test, *P* = 0.84, *n* ≥ 5) suggesting the possibility that it could reflect a different underlying molecular mechanism.

### Gating currents in WT CNGA1 channels

**Figure 11:**
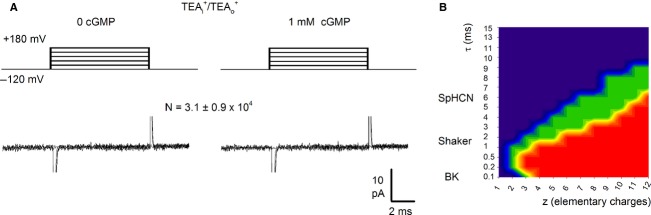
Gating currents measurements in WT CNGA1 channels. (A) Gating currents measurements in the presence of symmetrical TEA^+^ (110 mmol/L) and in the absence (left panels) and presence (right panels) of 1 mmol/L cGMP from a representative membrane patch, containing ≈30,000 ion channels. Only fast (<300 *μ*sec) nonlinear capacitive artifacts were visible after P/−4 procedure, similar to those recorded in uninjected oocytes. Voltage commands P were as shown in the upper panels. Similar results were observed in five additional giant patches. (B) Contour plot depicting the predicted gating currents color-coded signal-to-noise ratio (S/*N* < 4, blue; 4 ≤ S/*N* ≤ 6, green, S/*N* > 6, red) as a function of *z* and gating currents time constant *τ* for a membrane patch containing approximately 3 × 10^4^ ion channels. Off-gating currents for a simple two state model representing two possible states of a voltage sensor (resting and activated) were simulated. S/N was measured at the peak of the simulated off-gating currents. Background r.m.s. was assumed to be 1 pA.

In Na^+^, K^+^, and Ca^2+^ channels, the motion of the S4 helix can be detected by measuring gating currents (*I*_*g*_). Therefore, we attempted to measure *I*_*g*_ in WT CNGA1 channels (Fig. 11).

Let us suppose we have *N* ion channels and for each of them, if *z* elementary charges (in units of *e*_*0*_ equal to 1.6 × 10^−19^ C) translocate across the membrane in the time *Δt,* a gating current *I*_*g*_ approximately equal to *ze*_*0*_*N/Δt* can be measured. In K^+^ channels *z* is around 12–13 (Aggarwal and MacKinnon 1996; Seoh et al. 1996) and *Δt* is about 10^−3^ sec, so that in excised patches containing 4–5 × 10^3^ channels *I*_*g*_ is about 7 pA. The determination of *I*_*g*_ requires the reduction in all contaminating currents, primarily ionic, and capacitive currents (Armstrong and Bezanilla 1977). A reliable measurement of *I*_*g*_ in CNG channels can be obtained only when *N* is at least 10^4^ as in experiments using cut-open voltage-clamp technique or in macro/giant membrane patches (Perozo et al. 1992; Hilgemann and Lu 1998). In order to rapidly change the ionic medium, instead of using cut-open oocytes, we opted for patch pipettes with a diameter of 10–20 μm, where *N* could be larger than 2 × 10^4^ (see Methods and Fig. 1) and cGMP can be added and removed within 10^−1^ sec.

In order to verify that our experimental conditions could measure *I*_*g*_, we expressed in oocytes the mRNA coding for HCN channels from the sea urchin sperm (spHCN). We measured *I*_*g*_ with an amplitude of 40–50 pA from excised patches containing spHCN channels (see Methods) with kinetics and voltage dependence similar to those already described (Fig. 2). The r.m.s. of our current recordings is about 1 pA (at a bandwidth of 10 kHz) and the lowest measurable *I*_*g*_ is 5 pA, that is, five times the r.m.s. Assuming that *I*_*g*_ has an exponential decay (*τ* is 1–2 msec) and if *N* is about 3 × 10^4^, *I*_*g*_ produced by a *z* as low as 2 could be detected (Fig. 11B). However, when the voltage was stepped from −120 to +180 mV only capacitive artifact lasting no longer than 300 μsec was recorded (Fig. 11A). Similar results were obtained in five additional giant patches containing at least 3 × 10^4^ WT channels both in the absence and in the presence of 1 mmol/L cGMP (Fig. 11A).Therefore, based on the number of CNG channels present in the membrane patch (see Methods), we estimated that in the WT channels, either the value of *z* is about or less than 2 or *I*_*g*_ is too slow – and cannot be resolved because of uncertainties and noise of the baseline (Fig. 11B). In all these circumstances, the voltage sensor in CNGA1 channels does not move as in K^+^ channels. As the voltage sensor of CNG channels is capable of sustaining voltage-dependent gating, (Tang and Papazian 1997; Xu et al. 2010) the voltage sensor in WT CNGA1 channels moves much less than in K^+^ channels, in agreement with the estimate of z lower than 2 obtained from measurements of gating currents and from the analysis of the voltage gating in Cs^+^, MA^+^, and DMA^+^.

## Discussion

The present manuscript, by using tail and gating current measurements, clarifies the role of the pore and of the S4 helix toward the asymmetries in the *I*–*V* relationship observed in symmetrical conditions with different ionic species. Voltage gating highly depends on the permeating ion: for small monovalent alkali cations, such as Li^+^, Na^+^, and K^+^ gating is weakly voltage dependent, but gating is progressively voltage dependent for larger alkali cations, such as Rb^+^ and Cs^+^ and for organic cations such as EA^+^ and DMA^+^.

Voltage sensing in the WT CNGA1 channels is the result of a translocation of the usual voltage sensor, constituted by the S1–S4 domains and by voltage-dependent rearrangements of charged and polar groups within the pore region, primarily of Glu363. Measurements of *I*_*g*_ indicate that the voltage sensor in CNG channels moves much less than in K^+^ channels. Let us see now more in detail how *P*_o_ and γ_sc_ depend on *V* and which molecular structures could underlie this voltage sensing.

### Dependence of *P*_o_ and *γ*_sc_ on voltage in alkali and organic cations

Analysis of tail currents demonstrates that gating of CNG channels is voltage dependent in the presence of larger monovalent alkali cations Rb^+^ and Cs^+^, and of a variety of organic cations such as MA^+^, DMA^+^, and EA^+^. By means of noise, tail and macroscopic current analysis, we show that in the presence of all these cations, *P*_o_ increases with voltage – although with different magnitude and steepness – while macroscopic currents might be either inwardly rectifying (Rb^+^ and Cs^+^) or outwardly rectifying (MA^+^, DMA^+^, and EA^+^). This apparent contradiction could be easily disentangled if it is considered that the final shape of the *I*–*V* and *G*(*V*)/*G*_+200_ relationship depends not only on *P*_o_, but on both *P*_o_ and *γ*_sc_. A well-established way to discriminate between the open-pore rectification (voltage-dependent changes in *γ*_sc_) and channel voltage gating (voltage-dependent changes in *P*_o_) is to use a proper sequence of voltage commands in order to induce tail currents. After a perturbation (sudden change in membrane potential) tail currents arise from the kinetic of ion channels redistribution toward a new equilibrium (Hille 1992). Thus, tail currents are thought to reflect changes in *P*_o_ from a previous condition of equilibrium, whereas changes in *γ*_sc_ are believed to develop almost instantaneously. It was therefore possible to show with appropriate voltage protocols that in the presence of Rb^+^ and Cs^+^ *γ*_sc_ decreases of ∼50% and ∼70% from −200 to +200 mV, whereas *P*_o_ increases of ∼25% and ∼120%, respectively. In the presence of Rb^+^ and Cs^+^ *γ*_sc_ decreases more powerfully with *V* then what *P*_o_ increases, while the opposite occurs in symmetrical conditions of MA^+^, DMA^+^, and EA^+^. Therefore, the *I*–*V* relationship is inwardly rectifying in Rb^+^ and Cs^+^ – being driven by changes in *γ*_sc_ – while it is outwardly rectifying in the presence of MA^+^, DMA^+^, and EA^+^ – being mainly driven by changes in *P*_o_.

### The S4 voltage sensor in CNG channels and K^+^ channels

CNG channels belong to the superfamily of K^+^ voltage-gated channels but in the presence of small alkali cations such as Li^+^, Na^+^, or K^+^ exhibit little inherent sensitivity to voltage (Kaupp and Seifert 2002; Matulef and Zagotta 2003; Craven and Zagotta 2006). Studies with chimeric channels where the S4 transmembrane segment or the CNG voltage sensor paddle have been transplanted into different K^+^ channels have led to the notion that CNG channels harbor a potentially functional voltage-sensing domain (Tang and Papazian 1997; Xu et al. 2010). In the light of current understanding of voltage sensor operation, two distinct although not mutually exclusive mechanisms, effectively disabling channel voltage-dependent gating, can be considered. First of all the S4 helix, although functional, might be partially or almost completely impeded in its movements by the specific surrounding protein scaffolding (Tang and Papazian 1997). Alternatively, while S4 could move as much as in K_v_ channels, a loose electromechanical coupling between the S1–S4 voltage-sensing domain and the pore gate could result in channels with an altered gating and a reduced voltage sensitivity (Lu et al. 2002). To discriminate between these two different basic mechanisms, we analyzed capacitative transients in the presence of nonpermeant ions, such as TEA^+^ and NMDA^+^, and the effect of charges neutralization in the S4 helix.

Voltage sensor movement can be detected as nonlinear capacitive transients – known as gating currents – caused by the rearrangement of its electrostatic charges with respect to the electric field (Armstrong and Bezanilla 1974). If it is assumed that S4 freely moves in CNG channels as it does in usual K_v_ channels, 12–13 *e*_0_ per channel are expected to translocate through the electric field (Schoppa et al. 1992; Aggarwal and MacKinnon 1996; Seoh et al. 1996). Gating currents measurements in CNG channels provided us an upper bound on the number of elementary charges associated with voltage sensor movements, indicating that no more than 2 *e*_0_ per channel traverse the electric field. These results strongly suggest that although potentially functional, the S4 helix is constrained in its operation by the surrounding protein environment.

The analysis of tail currents in the presence of a variety of permeant cations indicates that a residual motion of the S4 transmembrane segment may still contribute to voltage gating in CNG channels. Indeed, when the second Arginine in the S4 segment is neutralized, voltage gating in the presence of large permeant ions is altered (Marchesi et al. 2012). In the present manuscript, we show that an early voltage-dependent transition associated with channel gating is affected in the R2Q_WT tandem construct, leaving almost unaltered voltage gating above 100 mV (Fig. 10). These results suggested the intriguing possibility that the two voltage-dependent transitions observed in the *I*_t_(*V*)/*I*_+200_ relationship might be controlled by different underlying molecular mechanisms: the steeper Boltzmann component developing at hyperpolarized potentials (*z*_1_ = 1.70 ± 0.10; *V*_mid1_ = −88.36 ± 5.99) is likely associated with conformational rearrangements in the S4-type voltage sensor, whereas the second component occurring at depolarized potentials (*z*_2_ = 1.00 ± 0.13; *V*_mid2_ = 140.18 ± 5.97) might reflect an inherent pore voltage sensitivity, as recently suggested for some pore mutant channels (Martínez-François et al. 2009; Sauer et al. 2011).

Our results indicate that in the presence of physiological ions, such as Na^+^ and K^+^, the additive contributions of two distinct mechanisms are at the basis of the reduced voltage sensitivity in CNG channels: (i) a reduced motion of the S4 transmembrane segment, which could be restricted by specific molecular interactions with neighbor residues; (ii) an inefficient coupling between the voltage-sensing domain and the channel gate which is modulated by the permeant ion.

### Flexibility at the selectivity filter in CNG and K^+^ channels

**Figure 12:**
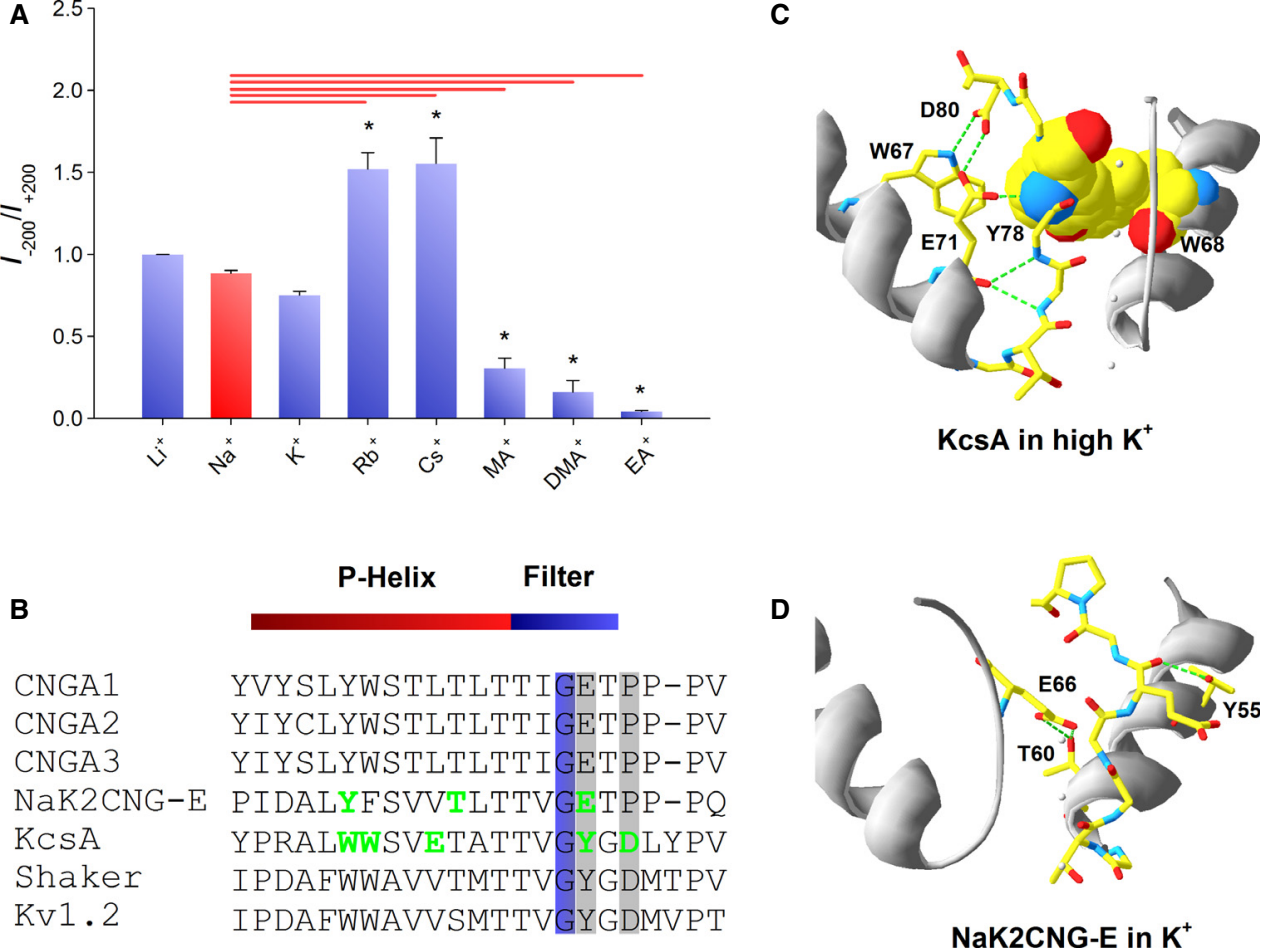
Partial sequence alignment among different ion channels and molecular structures of the KcsA and CNG mimicking NaK chimera featuring the interactions within the selectivity filter and surrounding scaffolding. (A) Dependence of *I*_-200_/*I*_+200_ on the permeating ions. ANOVA and Bonferroni post hoc tests were performed to compare each ion against Na^+^. Asterisks point out a statistical significance with a *P* < 0.01 (*n* ≥ 4.). (B) Partial sequence alignment in the pore region of the bovine alfa subunits (CNGA1-CNGA3), CNG mimicking NaK chimera (NaK2CNG-E) and K^+^ channels (KcsA, Shaker, Kv1.2). The conserved Glycine residue in the selectivity filter is highlighted in blue. The Tyrosine of the K+ selective channels signature sequence is replaced by a ring of Glutamate residues in CNG channels, while the aspartate immediately following the filter is substituted by a proline; highlighted in light gray. Residues involved in H-bonding in the KcsA and CNG chimera structures are marked in green. (C, D) Partial molecular structure of the KcsA channel (C) and NaK2CNG-E channel (D) as deposited in the Protein Data Bank (PDB entry code 1K4C and 3K0D, respectively) featuring the selectivity filter and the P-helix in two adjacent subunits. Crucial intersubunit and intrasubunit H bonds stabilizing the selectivity filter architecture are shown in both structures as dashed green lines. Residues side chains involved in H bonds are shown in ball-and-stick format while aromatic residues constraining the pore structure are shown in space-filling format. The K^+^ ions in the selectivity filter are shown as white spheres. The selectivity filter of the NaK2CNG-E chimera is stabilized by fewer hydrogen's bonds compared to KcsA channel.

Although several studies have examined voltage gating in CNG channels and the linkage between gating and permeation (Karpen et al. 1988; Benndorf et al. 1999; Gamel and Torre 2000; Holmgren 2003; Kusch et al. 2004; Nache et al. 2006; Martínez-François et al. 2009), the relation between the permeation-gating coupling and voltage has so far been neglected. In the present manuscript, we show that membrane voltage powerfully controls gating and permeation properties in CNG channels (Fig. 12A). Although novel, these findings are not entirely unexpected in the light of the so far available structural data and basic differences in the amino acidic sequences among the K+ and CNG channels (Fig. 12B). A comparison between the crystal structure of KcsA (Fig. 2C) and the CNG NaK chimera (Fig. 12D) shows that the KcsA selectivity filter is stabilized by extensive hydrogen bonding and Van Der Waals interactions with the pore helix, while this network of interactions is less developed in CNG channels (Fig. 12C and D). Indeed, the ring of Tyrosines which is thought to provide the putative rigidity and the network of hydrogen bonding (Fig. 12C) necessary to stabilize the structure of K+ channels selectivity filter (Doyle et al. 1998; Zhou et al. 2001; Bernèche and Roux 2005; Sauer et al. 2011) is replaced by a ring of Glutamates in CNG channels (Fig. 12B and D). Moreover, a wealth of crystallographic, electrophysiological, and MD simulations data indicate that in K^+^ channels one of the microscopic factors influencing pore stability is the occupancy of specific cations binding sites within the selectivity filter (López-Barneo et al. 1993; Shrivastava and Sansom 2000; Loboda et al. 2001; Zhou et al. 2001; Bernèche and Roux 2005; Piskorowski and Aldrich 2006). In this view, it is conceivable that the contribution of a permeating ion to the overall filter stability is enhanced in ion channels where the selectivity filter is loosely attached to the surrounding structures, establishing the molecular rational for the gating-permeation coupling. Membrane voltage, acting on specific charged and polar residues within the pore, is expected to further tune this coupling.

Over the last two decades, several functional studies have documented the linkage between gating and permeation in CNG channels (Ruiz and Karpen 1997; Hackos and Korenbrot 1999; Gamel and Torre 2000; Holmgren 2003; Kusch et al. 2004). For instance, it has been shown that ions that pass more slowly through CNG channels stabilize the open state of the channels (Kusch et al. 2004). We have recently shown that this linkage is powerfully controlled by voltage; the low-conductance long-lasting openings observed in symmetrical Rb^+^ and Cs^+^ single-channel recordings at positive voltages are readily converted into high-conductance flicker openings by membrane hyperpolarization (Marchesi et al. 2012). Interestingly, several evidences suggest that Glu363 is at the basis of these differences in ion conduction (Root and MacKinnon 1994; Marchesi et al. 2012). We believe that the heterogeneity of channel openings observed at hyperpolarized voltages arise from small conformational fluctuations of the selectivity filter which can be triggered by local rearrangements of Glu363 side chain. Because (i) the selectivity filter stability is expected to be sensitive to the local electrostatic as well as to the protonation state of ionizable groups (such as Glu363 side chain) and (ii) the main chain carbonyl oxygens interact directly with permeating ions, these conformational changes are expected to be strongly affected by the nature of the permeant ion, as well as local proton concentration and membrane voltage. In other words, the permeation of larger cations such as Rb^+^ and Cs^+^ unveils the operation of a pore voltage sensor controlling voltage-dependent asymmetries in single-channel conductance. The action of this pore voltage sensor is at the basis of the inward rectification in macroscopic currents here described when Rb^+^ and Cs^+^ permeate.

Thus, structural and electrophysiological evidence indicates that the selectivity filter of the CNG channels is an intrinsically dynamic structure possibly capable of fine structural rearrangements. This flexibility is necessary for the selectivity filter in order to act as the primary gate of the channel.
